# A Foldaxane‐Based Supramolecular Muscle‐Like Switch

**DOI:** 10.1002/open.202400076

**Published:** 2024-07-04

**Authors:** Philip Waelès, Frédéric Coutrot

**Affiliations:** ^1^ Supramolecular Machines and Architectures Team IBMM Université de Montpellier CNRS, ENSCM Montpellier France

**Keywords:** daisy chain, foldaxane, molecular muscle, supramolecular, template

## Abstract

[cn]daisy chain molecular muscle architectures are self‐assemblies of hermaphrodite monomers, which usually contain a macrocycle unit linked to a molecular thread that contains sites of interactions – *i. e*. molecular stations – for the macrocycle. In these multiply threaded structures, altering with control the affinity between macrocycles and stations allows for contraction and extension of the molecule, which is reminiscent of the operation of a muscle. Besides, the field that consists of combining helix and template‐containing rods to design foldaxane supramolecular assemblies is still underexplored. By using foldamer units as surrogates for macrocycles, Gan *et al*. reported the first supramolecular muscle‐like foldamer‐containing switch that can adopt, after chemical stimulus, either a contracted co‐conformational state or a degenerate‐like state for which a slow exchange occurred between the contracted and the stretched state.

## Graphical abstract

Quan Gan and co‐workers report the first supramolecular foldaxane‐based molecular muscle. The molecule is able to adopt a contracted state or a degenerate‐like state through the gliding of helixes between the contracted and stretched states, upon the invertible introduction of a bulky Fmoc group. Noteworthy, the supramolecular assembly is kinetically stable since its disassembly is much slower than gliding of the helix along the molecular rods.

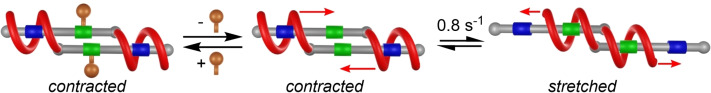

Among interlocked molecular architectures, [cn]daisy^[1]^ chain molecular muscles are appealing interwoven rotaxane oligomeric architectures that can adopt different co‐conformational states – from the contracted to the stretched state – upon chemical or photochemical stimuli. The overall chemical structure of each monomer that makes up the supramolecular assembly is qualified as hermaphrodite, because it consists of a macrocycle linked to a molecular thread that contains one or more molecular sites of interactions able to bind the inner of the macrocycle. In 2000, pioneering work in this field by Nobel laureate Jean‐Pierre Sauvage^[2]^ was reported and concerned the synthesis of the first linear [c2]daisy chain molecular muscle (Figure 1 a),^[3]^ and its operation through metal exchange. Contracted or stretched states of the rotaxane dimer could be obtained utilizing the respective preferential tetra‐ or penta‐coordination of copper^I^ or zinc^II^ for nitrogen ligands. It then took at least 8 years to see in the literature other singular linear [c2]daisy chain molecular muscles that operated, this time, through pH variations,^[4]^ light irradiation,^[5]^ solvent polarity changes^[6]^ or electrochemical redox reaction.^[7]^ In 2012, a cyclic [c2]daisy chain molecular muscle (Figure 1 b) was reported as an artificial sphincter mimic.^[8]^ This original structure was essentially based on the chemical connection of the two ends of a [c2]daisy chain in order to get a double‐lasso architecture that may tighten or loosen depending on pH. The efficient access to higher‐order [c3] and [c4]daisy chain architectures allowed to design new multiply‐interlocked molecular muscles that operate in two and three dimensions, respectively (Figure 1c–d).^[9]^

Now publishing in Angewandte Chemie, Quan Gan *et al*. reports a foldaxane^[10]^‐based supramolecular muscle, for which the foldamer unit is a surrogate unit for the macrocycle (Figure 1e).^[11]^

**Figure 1:**
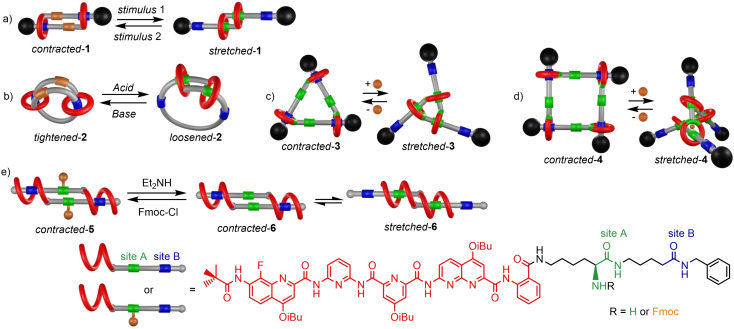
Cartoon representations of [cn]daisy chain‐based molecular muscles reported so far with their actuation; a) a stimuli‐responsive linear [c2]daisy chain molecular muscle; b) a pH‐responsive cyclic [c2]daisy chain molecular muscle; c) a metal‐dependent [c3]daisy chain molecular muscle operating in two dimensions; d) a metal‐dependent [c4]daisy chain molecular muscle operating in the three dimensions; e) current work showing a [c2]daisy chain‐like containing supramolecular muscle that holds helix units instead of macrocycles. For clarity, only the PP stereoisomer is represented for helix stereoisomerism.

The hermaphrodite monomer contains a well‐designed aromatic helix, which is covalently linked to a molecular rod that contains two amide sites of interactions – *i. e*. sites A and B – for the helix. Among them, an amide moiety – located at the *C*‐terminal side of a L‐lysine residue (site A, in green) – has been chosen as a switchable site of interaction. Indeed, the binding affinity between the helix and the amide of site A can be dramatically altered by the steric hindrance brought in the vicinity of site A, thanks to the possibility to incorporate and remove a hampering Fmoc protection on the free remaining α‐amino function.

Self‐assembly of the hermaphrodite monomer occurred in a hydrogen bond promoting solvent and resulted in the formation of a kinetically stable foldaxane dimer reminiscent of a [c2]daisy chain architecture. The presence of the hindering Fmoc moiety at binding site A sterically prevents from any association between the helix and the lysine amide, enforcing the helix to bind the sole amides of sites B and therefore conferring to the foldaxane dimer a contracted state (see compound *contracted*‐**5**). However, removing the Fmoc protection using diethylamine revealed sites A, as sites of interaction for the helix units that are no longer hindered. At this stage, the two sites of interaction A and B were both able to bind the helix with almost the same affinity. Hence, a degenerate‐like state occurred,^[12]^ in which the helix units slowly exchange between the two sites with a rate of 0.8 s^−1^. Then, in compound **6**, an equal proportion of the two contracted and stretched co‐conformational states was observed. Though slow, this exchange between translational isomers – from the contracted to the stretched isomer – proved to be much faster (7 order of magnitude) than the rate of disassembly of the supramolecular architecture. This rate comparison corroborates a muscle‐like gliding motion rather than an unfolding‐refolding process. Noteworthy, the process was invertible through the Fmoc reintroduction at the lysine amine function nearby site A, leading back to the *contracted*‐**5** co‐conformational state.

The attractive work by Gan *et al*. consists of a new step in the conception of the rare molecules that can adopt contractile properties. The access to this new sophisticated supramolecular architecture now paves the way to the design of new smart supramolecular material able to self‐assembly and operate upon orthogonal stimuli.

## Conflict of Interests

The authors declare no conflict of interest.

## Data Availability

Data sharing is not applicable to this article as no new data were created or analyzed in this study.
